# Editorial: Pharmacists and patient safety: focusing on drug safety

**DOI:** 10.3389/fphar.2026.1908184

**Published:** 2026-07-14

**Authors:** Zhiyao He, Wen Fang, Zhengyu Chen

**Affiliations:** 1 Department of Pharmacy, West China Hospital, Sichuan University, Chengdu, China; 2 Key Laboratory of Drug-Targeting and Drug Delivery System of the Education Ministry and Sichuan Province, Sichuan Research Center for Drug Precision Industrial Technology, West China School of Pharmacy, Sichuan University, Chengdu, China; 3 International Pharmaceutical Federation, The Hague, Netherlands

**Keywords:** drug safety, medication, patient safety, pharmacist, pharmacovigilance

## 1 Introduction

Patient safety is defined as “the absence of preventable harm to a patient and reduction of risk of unnecessary harm associated with healthcare to an acceptable minimum” by the World Health Organization (Aljaffary et al., 2022). Approximately 50% of medical harm worldwide occurs in primary care settings, of which about 80% can be effectively prevented through primary care services (Auraaen et al., 2018). Medication-related harm is considered preventable if it occurs as a result of an identifiable, modifiable cause and its recurrence can be avoided by appropriate adaptation to a process or adherence to guidelines (World Health Organization, 2024). Understanding the prevalence, nature and severity of preventable medication-related harm is critical for setting targets for clinically relevant, implementable improvements in patient safety (Panagioti et al., 2019; Hodkinson et al., 2020). To meet this challenge, the role of the pharmacist is undergoing a fundamental paradigm shift: transitioning from a traditional “drug supplier” to a “health guardian” deeply involved in clinical decision-making (Solh et al., 2026). The Research Topic “Pharmacists and patient safety: focusing on drug safety” aims to focus on patient safety by utilizing professional interventions, technological innovation, and evidence-based medicine to achieve precise prevention and control of medication risks and optimization of clinical outcomes.

## 2 Transformation of pharmacist roles and the value of professional interventions

Most general practitioners believe that pharmacists possess the clinical skills necessary to provide safe and effective care to patients (Sims and Campbell, 2017). A study led by Matej Stuhec et al. investigated the experiences of general practitioners (GPs), pharmacists, and patients regarding a pharmacist prescribing pilot project in Slovenia. All stakeholders held positive attitudes, with patients particularly appreciating the continuous monitoring provided by clinical pharmacists. Key barriers included a lack of legislative support, inadequate reimbursement mechanisms, and the need for structured educational training. The results support the phased implementation of dependent prescribing rights in Slovenia. Another study by the same group Stuhec et al. found that in the management of chronic disease patients—for instance, that after clinical pharmacists were authorized through a “Collaborative Practice Agreement” (CPA) to prescribe medications, adjust dosages, or discontinue inappropriate medications (deprescribing), the remission rate for conditions such as depression increased significantly from a baseline of 5%–76%. Furthermore, polypharmacy often led to increased healthcare costs, whereas professional intervention by clinical pharmacists significantly reduced potentially inappropriate medications and drug-drug interactions, which resulted in achieving cost savings of approximately EUR 456, 619 and yielding a remarkably high return on investment of about 22.3:1 (EUR 22.3 for every EUR 1 invested). A survey by Wrześniewska-Wal et al. in Poland showed positive attitudes toward pharmacist-led drug interaction reviews and instructions for new medications during the implementation of pharmaceutical care in community pharmacies. Support for pharmaceutical care was highest among respondents with chronic diseases, particularly diabetes. The results suggest combining pharmacist consultations with simple adherence-supporting tools. A cross-sectional study in Poland by Czekajewska et al. evaluated the attitudes of pharmacists and pharmacy students toward emergency contraception (EC) prescribing rights and the “conscience clause” (the right to refuse dispensing based on personal beliefs). The results showed that more than half (54.8%) of the respondents opposed granting pharmacists this right, with ethical concerns being more pronounced when dealing with patients under 18. The study found that individuals with stronger religious beliefs were more supportive of the clause, while those with higher levels of professional knowledge tended to oppose it.

Meanwhile, Qin et al. in China implemented a pre-audit system for inpatient medical orders based on the ORTCC (Objectives, Rules, Training, Checking, Culture) management model. Utilizing a “three-review and three-interception” mode, the system improved the qualification rate of medical orders from 76.38% to 97.69%, effectively intercepting errors in administration routes and frequencies to ensure patient medication safety. A pharmacist team led by Li et al. in a Chinese tertiary hospital developed the Pharmacist Intervention Evaluation (PIE) system, which achieved a classification efficacy significantly higher than the internationally used PCNE system (92.06% vs. 73.10%) by refining “severity” and “problem source”. This refined management enables more precise identification of physician decision-making errors and prescription/dispensing errors, thereby enhancing the quality management of pharmaceutical care. Simultaneously, the application of the PDCA (Plan-Do-Check-Act) cycle led by Li et al. in neurosurgery demonstrated excellent governance capabilities. Through multidisciplinary intervention, the rational prophylactic use rate of sodium valproate increased significantly from 32% to 93.41%. Additionally, the average course of treatment and Defined Daily Doses (DDDs) for injectable 5PA decreased substantially, effectively optimizing the utilization of medical resources.

Research in Saudi Arabia revealed deficiencies in healthcare professionals' knowledge of drug-food interactions (DFI) and linezolid interactions, calling for strengthened targeted training. A cross-sectional study by Shalali et al. revealed significant flaws in knowledge among Saudi healthcare professionals regarding DFI, with 60% of respondents having limited relevant knowledge. Only 19% had ever reported a DFI-related adverse event. Although most were aware of the interaction between linezolid and serotonergic drugs, a study by Aldairem et al. found that in-depth clinical knowledge (such as washout periods and diagnostic criteria) remained scarce. Only 10.3% of respondents demonstrated high levels of safe practice. A scoping review by Alanazi et al. showed that hospital pharmacists in Gulf Cooperation Council countries have varying levels of knowledge and non-standardized practices regarding the dispensing and counseling of oral anticoagulants (OAC).

## 3 Precision safety management and risk prevention in complex therapies

The professional value of pharmacists is increasingly prominent in intensive care and complex treatments (Wei et al., 2024). Pharmacists play an irreplaceable role in safeguarding patient safety through precision risk assessment, individualized medication monitoring, and multidisciplinary collaborative management.

The use of immune checkpoint inhibitors (ICI) has led to adverse reactions, including a significantly increased risk of tuberculosis (TB) infection and drug-class-specific digestive system inflammatory reactions. A real-world observational study by Zhan et al. analyzed 8,199 cancer patients treated with ICIs; the incidence of TB after treatment was 1.96%, significantly higher than that of the general population. The study emphasizes that in areas with a high burden of TB, systematic TB screening should be conducted before starting ICI therapy. An analysis of the FDA Adverse Event Reporting System (FAERS) database and a systematic review by Zou et al. revealed class-specific digestive toxicities for ICIs. PD-1 inhibitors are most strongly associated with inflammation of the upper gastrointestinal tract (esophagitis, gastritis) and hepatobiliary system (hepatitis, cholangitis), while CTLA-4 inhibitors (such as ipilimumab) show stronger signals for enterocolitis/colitis. These findings assist clinicians in personalized risk assessment and monitoring. Due to the inherent biases, lack of causality assessment, and data incompleteness in FAERS database analyses, misinterpretations of false-positive results or specific high-risk populations frequently occur (Hammad et al., 2025). Therefore, integrating a systematic review to perform data synthesis and decomposition ensures that clinical evidence is reliable and comprehensive.

Pediatric patient groups vary widely in age, body size, and developmental stage, particularly those in pediatric intensive care who present with various underlying diseases accompanied by changes in organ function (Teela et al., 2023). A study by Qin et al.analyzed cases of acute kidney injury (AKI) caused by vancomycin in a pediatric intensive care unit (PICU). The results showed that off-label high-dose use (>40 mg/kg/day) is closely related to the risk of renal injury. By implementing therapeutic drug monitoring (TDM) and Bayesian individualized dosing regimens, the AKI incidence rate dropped to zero, and the monitoring rate increased from 53.8% to 88.4%. A meta-analysis by Ma et al. further confirmed that pharmacist participation in vancomycin TDM not only increases the target blood concentration attainment rate by 60% but also significantly reduces the 30-day mortality and AKI risk for patients. Zhou et al. analyzed 94 children with infantile epileptic spasm syndrome (IESS) treated with Adrenocorticotropic hormone. The study confirmed that ACTH-induced hypertension is clearly dose-dependent, with the risk significantly correlated with the initial and daily average doses, highlighting the central role of initial dose selection in safe medication use. Furthermore, longer treatment durations and higher cumulative doses were positively correlated with the overall incidence of adverse reactions.

Perioperative optimization can effectively reduce postoperative complications. A randomized controlled trial by Guan et al. showed that in hysteroscopic surgery, adding low-dose mivacurium to the anesthesia induction regimen significantly improved the first-attempt success rate of laryngeal mask airway (LMA) insertion (98.8% vs. 48.2%). Additionally, it reduced the consumption of intraoperative anesthetics and lowered the incidence of adverse reactions such as postoperative sore throat (POST), nausea, and dizziness. A study by Seidenberg et al. found that scheduled administration of metamizole after cardiac surgery significantly reduced opioid consumption and improved pain scores without increasing the risk of renal injury. Wang et al. utilized liposomal bupivacaine for erector spinae plane block, providing long-lasting analgesia for spinal surgery, significantly reducing postoperative opioid consumption, and enhancing the quality of patient recovery.

## 4 Data-driven strategies and technology-enabled clinical decision support

The use of advanced technical means to process real-world data provides smarter assistance for clinical practice (Wu et al., 2026). Pharmacists leverage artificial intelligence, big data analytics, and advanced molecular diagnostic technologies to provide an evidence-based foundation for early treatment, mitigate the occurrence of adverse drug events, and safeguard patient medication safety.

He et al. demonstrated how prediction technology can shift from “post-event alertness” to “pre-event prevention”. This study utilized machine learning to establish a venous thromboembolism (VTE) risk prediction model for lymphoma patients. The optimal simplified model includes nine key predictors, among which venous catheterization, D-dimer, and lactate dehydrogenase (LDH) are positively correlated with VTE risk. This model (AUC = 0.954) can effectively identify high-risk patients, providing a basis for early prevention and anticoagulation strategies. A narrative review by Ye et al. explored the role of shared clinical decision support (SCDS) in enhancing patient safety. SCDS combines technology-generated recommendations (such as pharmacovigilance alerts) with a shared decision-making (SDM) framework, aiming to empower patients and reduce preventable adverse drug events (ADEs). A research report by Liu et al. described the treatment of a patient with pneumoconiosis and long-term corticosteroid use who developed a brain abscess due to *Nocardia farcinica* infection. After the patient experienced Grade III gastrointestinal toxicity with high-dose TMP-SMX, the medical team down-titrated the dose from 15 mg/kg/d to 7.5 mg/kg/d as a “bridge” therapy. Combined with imipenem and amikacin and early metagenomic next-generation sequencing diagnosis, clinical improvement was observed. Through “toxicity-guided” dose reduction and multi-drug combinations, the pharmacist assisted the medical team in successfully alleviating lethal toxicity while ensuring efficacy, providing a reference for clinical decision-making when standard doses cannot be tolerated in severe drug-resistant infections. A study by Yu et al. used text mining techniques (such as TF-IDF, LDA, and BERTopic) to analyze popular science articles on drug knowledge from WeChat public accounts. The study found that the five topics the public is most concerned about are: drug dosage, side effects, children’s infections, the efficacy of traditional Chinese medicine, and administration routes. Public interest is shifting from “side effects” to practical “medication issues,” providing creative guidance for the transformation of pharmacy professionals.

## 5 Quality verification of clinical evidence and the balance of socio-economic value

Pharmacists assess the merits of treatment regimens from scientific and economic perspectives (Sholihah et al., 2025). Pharmacists provide patients with treatment regimens that optimize both efficacy and safety across key areas, including administration route optimization, complex therapy selection, and high-risk medication monitoring.

A team led by Roccetti et al. cited a study correcting the false signal of “vaccine-induced vitiligo”, demonstrating that age-standardization analysis quantified and excluded structural selection bias, maintaining the authenticity of scientific conclusions. A retrospective real-world study by Chen et al. analyzed 609 patients treated with intravenous immunoglobulin (IVIG). The study found the incidence of thromboembolic events (TEE) to be 9.36%. Independent risk factors included: age ≥60 years, immobility ≥3 days, cumulative IVIG dose >100g, elevated pre-treatment D-dimer, and the use of glucocorticoids. The study suggested the prophylactic use of low-molecular-weight heparin for such high-risk populations, providing an evidence-based foundation. Zeng et al. used subcutaneous trastuzumab as an example to show that it is worth promoting due to its time-saving and cost-advantageous nature while having comparable efficacy. Meta-analysis showed that subcutaneous (SC) and intravenous (IV) trastuzumab are basically equivalent in efficacy (pathological complete response rate) and safety (serious adverse reactions). Patients significantly preferred subcutaneous administration (OR 63.02), primarily because it is more convenient and saves time. Cost analysis showed that the SC regimen has a cost advantage under the Chinese healthcare system. Rosas Sánchez et al. argued that generic drugs are key tools for solving the mental health crisis and reducing treatment costs. Generics are bioequivalent to original drugs but are typically more than 80% cheaper. The article pointed out that overcoming the psychological bias of doctors and patients toward the quality of generic drugs (such as the nocebo effect) is crucial for improving treatment adherence and health equity. Kang et al. compared second-line therapies for advanced liver cancer through a network meta-analysis, identifying the drug regimens that achieved the best balance between efficacy and tolerance. The results showed that Pembrolizumab and Ramucirumab achieved the best balance between efficacy and safety (lower incidence of Grade 3 or higher adverse reactions). Apatinib and Cabozantinib performed excellently in progression-free survival (PFS), but their toxicity was relatively high.

The themes of this issue collectively constitute a comprehensive map of medication safety management: from the macro transformation of pharmaceutical care models to the micro-optimization of individualized medication regimens; from cutting-edge machine learning predictions to basic clinical evidence quality control. The ultimate goal is to build a safer, more efficient, and more socially equitable medical environment.
